# Editorial: Parasite Infections: From Experimental Models to Natural Systems

**DOI:** 10.3389/fcimb.2018.00012

**Published:** 2018-02-02

**Authors:** Toni Aebischer, Kai Matuschewski, Susanne Hartmann

**Affiliations:** ^1^Unit 16 Mycotic and Parasitic Agents and Mycobacteria, Robert Koch-Institute, Berlin, Germany; ^2^Department of Molecular Parasitology, Institute of Biology, Humboldt University, Berlin, Germany; ^3^Department of Veterinary Medicine, Institute of Immunology, Freie Universität Berlin, Berlin, Germany

**Keywords:** parasite, malaria, toxoplasmosis, giardiasis, helminth infections

The aim of this research topic is to illustrate the multidisciplinary approaches of modern parasitology. The motivation to study parasites and parasitism varies. In the case of human and animal parasites, research is often motivated by the tremendous health threats and socioeconomic burden they pose. For instance, *Plasmodium*, the causative agent of malaria, continues to be the most important vector-borne pathogen and was responsible for more than 200 million new cases and 400,000 deaths worldwide in 2016 (WHO, 2016a). Another example is soil-transmitted helminth infections affecting 24% of the world's population, primarily school children (WHO, 2017). Many parasites are etiologic agents of infections classified as Neglected Tropical Diseases (NTDs) and continue to afflict societies with limited resources (Hotez et al., 2007). Moreover, research on parasites of wildlife can be critical for understanding animal communities and disease ecology (Gomez and Nichols, 2013; Johnson et al., 2015) and—by extrapolation—ecosystems' dynamics.

Parasites are defined by life style but reflect polyphyletic groups of protozoa, helminthes, and arthropods. In investigating these non-model organisms, studies on parasites often fall short of in-depth molecular, genetic, or biochemical analyses that characterize investigations of established laboratory-adapted organisms. This research topic (RT) collates 20 contributions by >100 authors, which we introduce here briefly by classifying them according to four sections along the path from experimental models to natural systems:

## Experimental models: hypothesis testing, translation, and limits

Experimental models such as laboratory mice or model cell culture systems have the great advantage to mechanistically answer biological questions. This proven strategy is used by Ngwa and colleagues who used *in vitro* differentiation of *Plasmodium falciparum* gametocytes to describe epigenetic changes due to histone acetylation that are likely relevant during parasite transmission from human host to vector (Ngwa et al.). Interaction of parasite and host factors can also reliably be studied in experimental models. Here, Dunst and colleagues identified the microfilament tethering factor moesin as an interacting partner of *P. falciparum* GPI purified from blood stages parasites (Dunst et al.). The authors show that absence of moesin influences neither life cycle progression nor malaria-related pathology, calling for further studies to identify GPI-binding recognition factors in the host. Analyses in mouse parasites illustrate key elements of the tool-boxes available to parasitological research in experimental model organisms.

This tool-box is also exploited in the contribution by Souza et al.. They apply a murine model of microbial sepsis to test the collateral damage by a pro-inflammatory immune response elicited by a persisting preexisting *Toxoplasma gondii* infection. The study on neutrophils in cerebral inflammation due to *Toxoplasma* infection contributed by Biswas and coworkers highlights another benefit of using laboratory mouse models, namely the broad range of antibodies available to identify and characterize specific immune cell subsets, based on signature proteins and cytokines (Biswas et al.). This allowed the authors to show that two distinct populations of inflammatory neutrophils act together to diminish parasite infiltration into the brain and reduce experimental cerebral toxoplasmosis.

Deciphering of immune cell interaction and cellular networks of host responses to parasites often rely on laboratory models. In this RT Bock and colleagues define so-called Th2/1 hybrid cells that combine lineage transcription factors and cytokine expression patterns of CD4^+^ Th1 as well as Th2 cells as integral parts of the immune response to nematodes (Bock et al.). Along the same line, Ahmed and colleagues analyze changes of the nematode-specific T cell response in a coinfection setting with an opposing pathogen (Ahmed et al.). In the helminth field the vast possibilities of working with the free-living model nematode *Caenorhabditis elegans* are often exploited to extrapolate findings to parasitic nematodes. Here, Midha et al. review the current knowledge and potential new directions to study reciprocal interactions of nematodes with their microbial environment (Midha et al.).

## Appraisals of experimental models

Cell culture and animal models for parasitic diseases often remain suboptimal, and their correlation with the infection of the host species of interest is a frequent matter of debate. Dunst and coworkers review present knowledge on the relevance of cytokines in the etiology of cerebral malaria (Dunst et al.). Although the respective murine model can only offer an incomplete assessment of this major complication of human malaria, it permits the fine resolution of molecular mechanisms involved in the development of cerebral malaria. Dunst et al. integrate findings from the murine experimental malaria model with *in vitro* observations and results of clinical studies to deduce a potential sequence of pathophysiological events that entails the activation of endothelial cells and leukocyte recruitment and ultimately leads to permeability of the blood-brain barrier and neuroinflammation. Acknowledging the difficulties to establish a suitable model host for human *P. falciparum*, Cunha and colleagues present a Chlodronate-liposome-based protocol for new world common squirrel monkeys (*Saimiri sciureus*) to render these hosts susceptible to experimental *P. falciparum* inoculations by depletion of phagocytes (Cunha et al.). This might substitute the current common practice of surgical splenectomy.

*Giardia duodenalis* is a cause of chronic diarrheal disease globally, but not every infected host develops symptoms. Pathogenic mechanisms, such as the breakdown of intestinal barriers, have been primarily investigated using human intestinal cell lines, such as CaCo-2 cells. Kraft and colleagues attempt a critical analysis of the value of the latter as a model of pathogenesis based on literature and own observations (Kraft et al.). They propose that this set up rather models asymptomatic colonization and may serve to screen for till date elusive non-host, non-parasite factors precipitating disease.

An appraisal of limitations and pitfalls, underexplored virtues and promises of small rodent models is contributed by Ehret and colleagues. They review illustrative examples and discuss the translational potential of non-standard rodent resources, such as collaborative crosses of *Mus musculus*, humanized mice, and wildtype rodents (Ehret et al.).

## Natural systems

### Technological advances for investigating natural parasite-host systems

A prerequisite to study cognate parasite-host systems in non-model situations is proper identification of parasite species. DNA sequencing at low cost is enabling encyclopedic projects like the barcoding of all life (Blaxter, 2016; Hebert et al., 2016). Similarly, mass spectrometry-based species identification has also come of age also for pathogen identification, particularly in medical diagnostics (Greco and Cristea, 2017). Murugayan and Roesler review recent advances in this approach to identify pests, parasites, and vectors (Murugayan and Roesler). In their contribution, Bredtmann and colleagues compare barcoding and MS-based approaches and discuss their combination to solve the long pending issue of species differentiation of cyathostomine nematodes that parasitize horses (Bredtmann et al.). Heitlinger et al. report on their use of multi-amplicon sequencing to assess the load of different gastrointestinal parasites in free-living spotted hyenas (*Crocuta crocuta*) (Heitlinger et al.). Together with long-term data on these social animals their results suggest a higher diversity of eukaryotes, which include presumed parasitic taxa, in the intestine of high-ranking animals that usually exhibit a higher Darwinian fitness.

Modeling infectious processes is a highly dynamic field, and Ribeiro et al. contributed a report of their cellular automata/lattice gas modeling strategy to simulate immune cell-parasite interaction in cutaneous leishmaniasis based on ecto-nuclease levels expressed by different *Leishmania* spp. (Ribeiro et al.). In general, it is probably the combination of modeling and technological advances that holds promise for exciting research directions in wildlife parasitology.

### Studies in natural systems

Several contributions addressed their parasitism-related research questions directly in the natural hosts. Jennes and co-workers study the response to *T. gondii* infections in pigs (Jennes et al.). *Toxoplasma*-infected pork meat is a major source driving the epidemiology of toxoplasmosis, for example in Germany (Wilking et al., 2016). Jennes et al. propose a strategy to reduce parasite burden in pork meat based on data from an epidemiologically relevant host—parasite pair of this zoonosis.

Zoonotic parasites of livestock are extremely relevant for global health and global food security. So is the good health of honey bees (*Apis mellifera*), which has direct impact on pollination efficacy and honey production. Gisder and colleagues contributed a longitudinal analysis of *Nosema apis* and the emerging *N. ceranae* prevalences in honey bees of North East Germany (Gisder et al.). Although infestation with *N. ceranae* has been linked to alarming colony losses in bee populations Gisder et al. find no evidence for a general fitness advantage of *N. ceranae* in their survey data. They propose climate-dependent relative advantages of one over the other *Nosema* species and show in an insect host cell model that *N. ceranae* has higher replication capacity than *N. apis* when temperatures are elevated.

While bees may classify as lifestock, *Anopheles* mosquitoes are best known as vectors of diverse pathogens. A key pathway governing their own immune response to infection is reviewed by Zakovic and Levashina who summarize knowledge on the role of the mosquitoe's NF-kB-like signaling pathway REL2 in the response to plasmodial infection (Zakovic and Levashina). Understanding these aspects nurture the hope for new avenues in vector-centered malaria management.

## The urgent need for better models and methods in natural parasite systems

Arguably, instructive models to test novel malaria vaccine candidates would be of very high translational relevance. A vaccine to prevent human malaria is WHO's target by the year 2030 (WHO, 2016b). Two contributions to this RT discuss malaria vaccines. Jaurige and Seeberger review examples, strategy and status of vaccines consisting of carbohydrate antigens against malaria, but also against toxoplasmosis and leishmaniasis (Jaurige and Seeberger). The idea of a live attenuated vaccine has a long history in malaria research. Irradiated or chemically attenuated parasites have been used but may not be the ultimate solution. In that context, Kreutzfeld and coworkers discuss the promise that genetically attenuated *Plasmodium* spp., which do not develop beyond the liver stages, hold with respect to inducing a protective immune response (Kreutzfeld et al.). They conclude that realizing the potential of such parasites requires earnest investment into murine models to improve their relevance for translation.

We hope that this RT sparks interest for parasitic infections, which remain a research priority in medicine, veterinary medicine, and public health.

## Author contributions

SH, TA, and KM were the three editors of the research topic and thus put together the editorial for the RT “Parasite Infections: From experimental models to natural systems.”

### Conflict of interest statement

The authors declare that the research was conducted in the absence of any commercial or financial relationships that could be construed as a potential conflict of interest.

## References

[B1] Blaxter M. (2016). Imagining Sisyphus happy: DNA barcoding and the unnamed majority. Philos. Trans. R. Soc. B 371:20150329. 10.1098/rstb.2015.0329 27481781 PMC4971181

[B2] Gómez A. Nichols E. (2013). Neglected wild life: parasitic biodiversity as a conservation target. Int. J. Parasitol. Paras. Wildl. 2, 222–227. 10.1016/j.ijppaw.2013.07.002 24533340 PMC3862516

[B3] Greco T. M. Cristea I. M. (2017). Proteomics tracing the footsteps of infectious disease. Mol. Cell. Proteomics 16, S5–S14. 10.1074/mcp.O116.066001 28163258 PMC5393401

[B4] Hebert P. D. Hollingsworth P. M. Hajibabaei M. (2016). From writing to reading the encyclopedia of life. Philos. Trans. R. Soc. B 371:20150321. 10.1098/rstb.2015.0321 27481778 PMC4971178

[B5] Hotez P. Molyneux D. Ferwick A. Kumaresan J. Sachs S. E. Sachs J. D.. (2007). Control of neglected tropical diseases. New Engl. J. Med. 357, 1018–1027. 10.1056/NEJMra064142 17804846

[B6] Johnson P. T. J. de Roode J. C. Fenton A. (2015). Why infectious disease research needs community ecology. Science 349:1295504. 10.1126/science.1259504 26339035 PMC4863701

[B7] WHO (2016a). World Malaria Report 2016. Geneva: WHO.

[B8] WHO (2016b). Tables of Malaria Vaccine Projects Globally: The Rainbow Table. Geneva: WHO.

[B9] WHO (2017). Fact Sheet: Soil-Transmitted Helminth Infections. Geneva: WHO.

[B10] Wilking H. Thamm M. Stark K. Aebischer T. Seeber F. (2016). Prevalence, incidence estimations, and risk factors of *Toxoplasma gondii* infection in Germany: a representative, cross-sectional, serological study. Sci. Rep. 6:22551. 10.1038/srep22551 26936108 PMC4776094

